# Position-invariant icon remapping facilities search performance in foldable smartphones through the contribution of contextual cueing

**DOI:** 10.1186/s41235-025-00668-9

**Published:** 2025-09-01

**Authors:** Yuzhu Ji, Yubing Wang, Wenjing Jin, Haiyang Jin, Weidan Xu, Hongting Li

**Affiliations:** 1 College of Education, Zhejiang University of Technology, Hangzhou, China; 2 Department of Psychology, Zhejiang Sci-Tech University, Hangzhou, China; 3 School of Business Administration (MBA), Zhejiang Gongshang University, No.18, Xuezheng Str., Qiantang District, Hangzhou, 310018 China

**Keywords:** Foldable devices, Icon remapping, Contextual cueing, Visual search, Usability

## Abstract

Enabling smartphones to be foldable provides an effective approach to achieving both portability and large screens. Notably, switches between closed and open states in using foldable smartphones are accompanied by icon remapping, which can decrease smartphone usability if it fails to match usage expectations. This study conducted two experiments to examine the usability of two popular icon remapping methods, position-invariant and order-invariant, as well as the specific roles of contextual cues in different icon remapping. Results revealed that position-invariant remapping is more effective in terms of usability with faster searching speed. Also, getting familiar with the spatial arrangement of icons on small screens reduced performance differences between the two remapping methods on large screens while improving search speed only on small screens. These results together suggest that position-invariant remapping is a more effective design, highlighting the significance of contextual cueing in optimizing icon remapping designs in foldable smartphones.

**Supplementary Information:**

The online version contains supplementary material available at 10.1186/s41235-025-00668-9.

## Significance statement

Incorporating cognitive science principles into interface design offers valuable insights for enhancing real-world user experiences. In recent years, foldable smartphones have emerged as a rapidly growing trend in mobile technology, introducing unique interaction challenges that impact usability. Unlike conventional devices, screen transitions in foldable phones (between closed and open states) involve simultaneous changes in screen size and the number and spatial arrangement of icons, placing greater demands on users’ visual attention. Despite their increasing popularity, it remains unclear which icon remapping method best supports efficient search performance during screen transitions and why certain designs are more cognitively effective than others. We compared two common icon remapping methods and examined the underlying cognitive mechanisms involved. Our findings demonstrate that position-invariant remapping is the preferred design, as it preserves more horizontal and vertical spatial relationships between icons (i.e., contextual cues), enhancing search performance and helping maintain usability during screen transitions. The findings advance our understanding of visual search while providing practical insights for designing more intuitive and user-friendly interfaces.

**Supplementary Information:**

The online version contains supplementary material available at 10.1186/s41235-025-00668-9.

## Introduction

In recent years, the increasing screen size in mobile smart devices has become a key feature in product evolutions. As screen sizes continue to grow, the foldability of smartphones offers both immersive large-screen experiences and portability. The large screen in its open state not only provides more refined and captivating video and image effects but also enables new multitasking experiences through features like split-screen operations, thereby offering a richer and more personalized human–computer interaction experience. However, when a foldable smartphone switches between small and large screens (i.e., between its closed and open states), the icons are rearranged, and the spatial relationships between icons are altered. These alternations may decrease smartphone usability if they fail to match the expectations or experiences of users. As shown in previous research on icon remapping between tablet orientations (Shi et al., 2013), icon remapping could reduce the effectiveness of contextual cueing, resulting in lower search performance. Unlike tablets, where switches between landscape and portrait modes only alter orientations but not the screen size, switches between closed and open states in foldable smartphones are accompanied by changes both in screen sizes and icon numbers. Therefore, state switches in portable smartphones likely involved distinct cognitive mechanisms for icon remapping. Nevertheless, there is limited research on how different remapping designs affect icon search performance in foldable smartphones. Investigations into this aspect will bring valuable insights into how people deal with icon remapping in maximizing the usability of foldable smartphones and provide practical suggestions for smartphone manufacturers to optimize the designs to increase product usability.

One critical factor that aids in the search for icons in mobile devices is contextual cueing, which refers to the facilitation of invariant distractor configurations in searching for targets (Chen et al., 2024; Chun & Jiang, 1998; Geyer et al., 2010, 2024; Peterson et al., 2022; Wolfe & Horowitz, 2017). In the classic paradigm, participants were instructed to search for a target letter among distractor ones. In the old display condition, the target letter was shown in an invariant and repeated spatial arrangement of distractors. In contrast, in the new display condition, the target letter was among distractors with a randomly compiled arrangement. Results revealed that the response times were shorter in the old compared to the new displays, showing a cueing effect (Chun & Jiang, 1998). The global context of repeated spatial configurations guided the attention to the target location efficiently and, therefore, led to the contextual cueing effect, even when participants were unaware of the repetition (Goujon et al., 2015; Sisk et al., 2019).

Learning of the context–target associations is fast and reliable (Zellin et al., 2014). It has been consistently observed in various scenarios, such as when the target and distractors with the same arrangement (i.e., the whole display) were shifted along horizontal or vertical axes, or when the whole display was scaled into different sizes (Jiang & Wagner, 2004). The cueing effect was also found even when only half or one quadrant of the whole display items was repeated (Brady & Chun, 2007). A recent EEG study using a half-display repetition design also found that predictive contexts facilitated target detection on both sides (Chen et al., 2025b). These findings suggest that only partial repetitions of the whole display are sufficient to lead to increased target search performance (Brady & Chun, 2007; Chun & Jiang, 1998).

On the other hand, the contextual cueing effect is reduced if the relative positions between the target and other items are disrupted. Manginelli and Pollmann (2009) relocated the target in the whole repeated displays and observed a diminished cueing effect. This result was then replicated in later studies (Conci et al., 2011; Makovski & Jiang, 2010; Zellin et al., 2013). Moreover, Olson and Chun (2002) manipulated the distance between the target and the repeated distractor arrangements; they found that the increased distance decreased the contextual cueing effect. These findings continue to indicate that the positions of the target and distractors in the whole display are the key to the contextual cueing effect.

Research from both sides above suggests that the predictability of the target location based on the spatial arrangement of identical distractor-target configurations is critical for contextual cueing (Zinchenko et al., 2018, 2020b). This should also apply to searching for target application icons in mobile devices. In mobile devices, the predictability of target locations varies in different remapping methods used, which, therefore, should lead to distinctive contextual cueing effects. Shi et al. (2013) examined the contextual cueing in different icon remapping methods on tablets. They observed the contextual cueing effect only in the local-invariant and central-invariant remapping conditions, where the relative spatial relationships between the target and other items were preserved more. As such, the contextual cueing effect could be used to explain differences in search performance for different icon remapping designs in mobile devices, at least in tablets.

Nevertheless, the role of context cueing effect remains unclear in different icon remapping methods in foldable smartphones. Unlike tablets, switches between open and closed states in foldable smartphones not only change screen sizes but also lead to different numbers of displayed icons. Such switches increase the uncertainty in target-context relationships. Recent evidence suggests that reduced predictability of target locations may recruit alternative cognitive mechanisms such as distractor suppression. Thus, contextual cueing is not solely driven by predictability; rather, it may involve a dynamic balance between facilitation and suppression processes that depend on context uncertainty (Chen et al., 2024, 2025a, 2025b). This implies that both the usability and cognitive mechanisms underlying icon remapping in foldable smartphones may differ from those observed in traditional tablet interfaces.

**Fig. 1:**
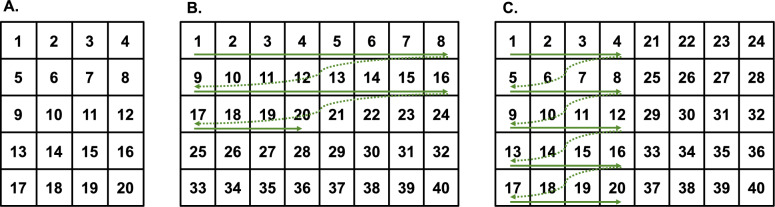
Schematic illustration of icon remapping. *Note*: **A** Small screen, i.e., screen in the closed state. **B** Large screen (i.e., screen in the open state), where icons were arranged with the order-invariant remapping. **C** Large screens (i.e., screens in the open state), where icons were arranged with the position-invariant remapping. Each number represents a specific icon. Real icons were used in the experiment

Currently, two main icon remapping methods have been implemented in foldable smartphones: order-invariant remapping and position-invariant remapping (see Fig. 1). This study systematically investigated the usability of these two remapping methods in foldable smartphones. Following previous research (Shi et al., 2013), the study employed a visual search paradigm, using real smartphone icons as search items. Icon search response times were used as the primary metrics for evaluating the usability of specific remapping methods.

The study consisted of two experiments. Experiment 1 examined the differences in search performance between the two remapping methods. Since position-invariant remapping minimally altered spatial arrangements, we expected that position-invariant remapping would yield better search performance compared to order-invariant remapping. Experiment 2 explored the role of contextual cueing in different remapping methods. We manipulated the cueing with learning, which is expected to reduce the differences between the two remapping methods.

## Experiment 1

### Method

#### Participants

Forty undergraduates (15 males, 25 females, age = 18 ~ 25) participated in Experiment 1. All participants had normal or corrected-to-normal vision and were right-handed, and they provided written consent forms before participating in the experiments. The local ethics committee approved this study.

A power analysis using G*Power software (Faul et al., 2007) indicated that a total of 40 participants was needed to detect a main effect of Remapping for the effect size of partial eta squares of 0.08 (Cohen’s *f* of 0.29), with a statistical power of 0.95 and an alpha level of 0.05. Therefore, we recruited 40 participants for this experiment.

#### Stimuli

Forty actual app icons used on real smartphones were employed in this experiment to increase ecological validity. Each icon extended 1.43° × 1.43°. To create screens for closed and open states in foldable mobiles, we make images for small and large screens with those actual icons. Specifically, the layout of small screens consisted of five rows and four columns (Fig. 1A), extending 7.15° × 8.77°. By contrast, the layout of large screens consisted of five rows and eight columns (e.g., Fig. 1B), doubling the number of columns and spanning a visual angle of 14.44° × 8.77°.

We created large screen stimuli with both order-invariant and position-invariant remapping. For order-invariant remapping (Fig. 1B), the order of icons on small screens was kept row-wise on large screens. For position-invariant remapping (Fig. 1C), the position of all icons on small screens remained constant on the left or right half of large screens.

As mentioned earlier, the size of large screens is two-fold that of small screens, whereby the icon number of large screens was doubled. As such, we considered two situations: (1) transitions between the first small screen (i.e., icon index 1–20, Fig. 1A) and the large screen; (2) transitions between the second small screen (i.e., icon index 21–40) and the large screen.

**Fig. 2:**
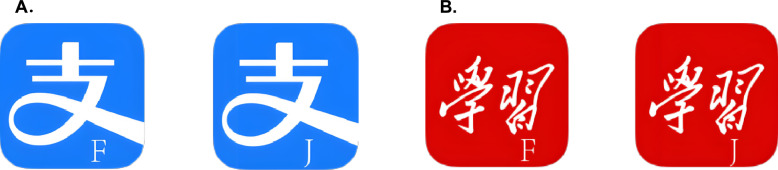
Search targets with response targets in Experiment 1. *Note*: The search targets with response targets for the first (**A**) and second (**B**) small screens. Participants were instructed to press the keys according to the response target (“F” or “J”) shown on the search target (the target icon)

The task in this experiment was to search for specific icons, i.e., the search target. In particular, we used the icons of"Alipay"and"Xuexi Qiangguo"(Fig. 2), two popular applications for participants, as search targets for the first and second small screens, respectively. To avoid participants making random responses without searching for the icons, we added response targets (i.e., letters “F” or “J”) in the bottom right corner of the search targets. We instructed participants to press the keys according to the response target shown on the search target.

#### Procedure

All stimuli were displayed on a 19-inch LED monitor (refresh rate 60 Hz, resolution 1440 × 900). The experiment was administrated with PsychoPy-2022 (Peirce et al., 2019).

There were four blocks: 2 (Transition: small-to-large vs. large-to-small) × 2 (Remapping: order-invariant vs. position-invariant), where the run order was fully randomized for each participant. Each block consisted of two mini-blocks. The two mini-blocks displayed the icons from the first and second small screens, respectively. At the beginning of each mini-block, the target icon was shown on the monitor, and participants were instructed to search for this target icon for all screens within this mini-block. Each mini-block consisted of 20 trials.

**Fig. 3:**
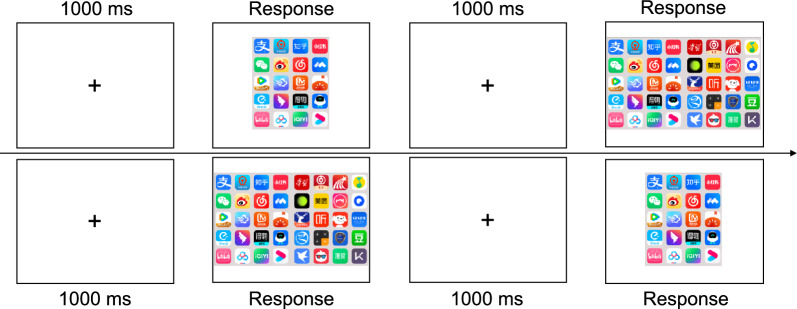
Trial procedure in Experiment 1. *Note:* The first row shows the trial procedure for small-to-large screen transition (i.e., from closed to open states in foldable phones), while the second row shows the large-to-small transition (i.e., from open to closed states in foldable phones)

Each trial (Fig. 3) began with an 1000 ms fixation cross, followed by a screen stimulus (e.g., a small screen stimulus in the small-to-large transition). The task was to search for the target icon, which was shown at the beginning of the min-block, and press the corresponding key as quickly and accurately as possible. Then, another 1000 ms fixation cross was shown, after which another screen stimulus was displayed (e.g., a large screen stimulus in the small-to-large transition). Participants were instructed to search for the same target icon and press the corresponding key as quickly and accurately as possible.

#### Statistical analyses

This experiment employed a 2 (Transition: small-to-large vs. large-to-small) × 2 (Remapping: order-invariant vs. position-invariant) within-subject design. As the main interest was to inspect the search performance for icons after remapping, we only analyzed the performance for large screens in small-to-large transitions and that for small screens in large-to-small transitions.

Jamovi 2.3.28 (The jamovi project, 2024) was used to conduct 2 × 2 repeated measures ANOVAs. Response times of all trials were above 200 ms, and therefore, no trials were excluded from the following analyses.

### Results

Overall, the average accuracies for each condition were all above 97%, suggesting that participants completed the task by following instructions. Because of the restricted range, accuracy was not further analyzed.

**Fig. 4:**
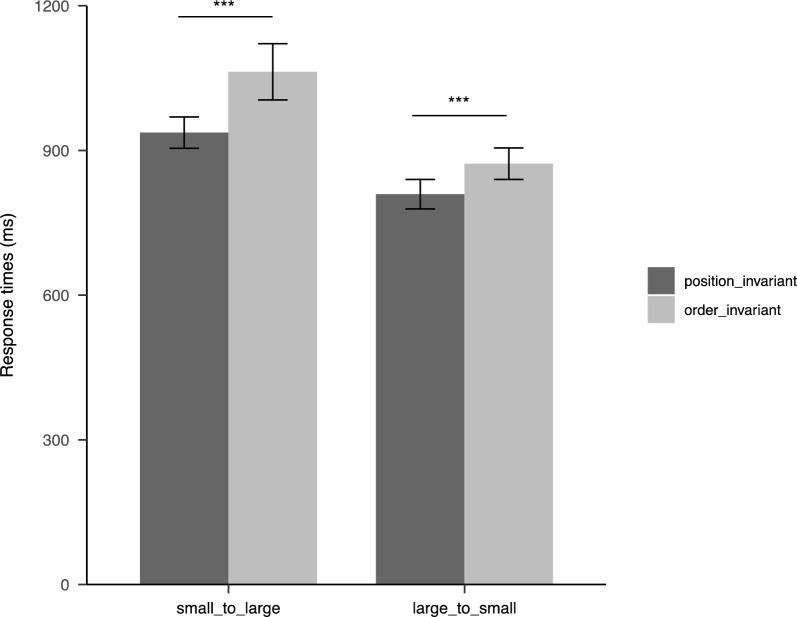
Results in Experiment 1. *Note:* Correct response times for searching target icons as a function of Transition (small-to-large vs. large-to-small) and Remapping (order-invariant vs. position-invariant). Error bars denote the 95% confidence intervals (CIs), ***p < 0.001

The following analysis only focused on correct response times (RT). A 2 (Remapping: order-invariant vs. position-invariant) × 2 (Transition: small-to-large vs. large-to-small) repeated measures ANOVA was conducted on correct RT (Table 1, Fig. 4). The results revealed the significant main effect of Remapping (*F*_1,39_ = 50.52, *p* < 0.001, η_p_^2^ = 0.564), with shorted RT in position-invariant remapping compared to order-invariant remapping. A significant main effect of Transition was also observed (*F*_1,39_ = 54.71, *p* < 0.001, η_p_^2^ = 0.584), with shorter RT in the large-to-small screen condition compared to the small-to-large screen condition. Additionally, the interaction between Remapping and Transition was significant (*F*_1,39_ = 7.32, *p* = 0.01, η_p_^2^ = 0.158). Simple effects analysis revealed that RT in position-invariant remapping was significantly shorter than for order-invariant remapping in both the small-to-large condition (*t*_39_ = − 5.77, *p*_bonferroni corrected_ < 0.001) and the large-to-small condition (*t*_*39*_ = − 5.23, *p*_bonferroni corrected_ < 0.001). The significant interaction suggests that the differences in search performance between the two remapping methods were greater in the small-to-large condition compared to the large-to-small condition.

**Table 1:** Descriptive statistics of correct RTs in experiment 1 (M ± SD)

Remapping method	Small-to-large (ms)	Large-to-small (ms)
Position-invariant	937 ± 102	809 ± 96
Order-invariant	1063 ± 182	873 ± 102

In addition, to inspect whether different remapping methods enhance search performance relative to a non-remapping baseline, we conducted a 3 (Remapping: order-invariant vs. position-invariant vs. non-remapping) × 2 (Screen: large vs. small) repeated-measures ANOVA. Results showed that, in short, position-invariant remapping outperformed order-invariant remapping and the non-remapping baseline. Detailed results are available in the Supplementary Materials.

### Discussion

In Experiment 1, participants performed a visual search task in different Remapping and Transition conditions. Results revealed that the RT for the position-invariant remapping was significantly shorter than that for the order-invariant remapping, supporting the experimental hypothesis. Notably, during the position-invariant remapping, the relative spatial arrangement of distractors and target icons remained unchanged, which likely elicited a stronger contextual cueing effect, helping participants predict the location of the target icon. If it is the contextual cueing that leads to better performance in the position-invariant remapping, we should also observe improved search performance when we enhance the contextual cueing, especially in the order-invariant remapping where the contextual cueing was weaker. Therefore, in Experiment 2, we instructed participants to learn the icon locations and then search for target icons to investigate further the role of contextual cueing in searching performance after icon remapping.

## Experiment 2

Compared to Experiment 1, Experiment 2 introduced an additional learning phase in which participants got familiar with the locations of certain target icons displayed on a small screen. This phase aimed to establish contextual cues related to the spatial arrangement of some icons prior to the test phase. Experiment 2 sought to examine whether the acquired contextual cues could enhance search performance following icon remapping.

### Method

#### Participants

Another group of 29 undergraduates (16 males, 14 females, age = 18 ~ 25) participated in Experiment 2. All participants had normal or corrected-to-normal vision and were right-handed, and they provided written consent forms before participating in the experiments. The local ethics committee approved this study.

An effect size of η_p_^2^ = 0.564 for the main effect of remapping was observed in Experiment 1. We expected that this effect size would be much larger than that of the main interest in Experiment 2. To additionally ensure sufficient statistical power, we employed 1/5 of η_p_^2^ = 0.564 as the assumed effect in power analysis (i.e., η_p_^2^ = 0.113/Cohen’s f = 0.36). By using this effect size together with an alpha level of 0.05, the power analysis conducted with G*Power software (Faul et al., 2007) shows that a total sample of 28 participants would provide a statistical power of 0.95. Therefore, the actual sample size of 29 participants in this study provided sufficient power.

#### Stimuli

**Fig. 5:**
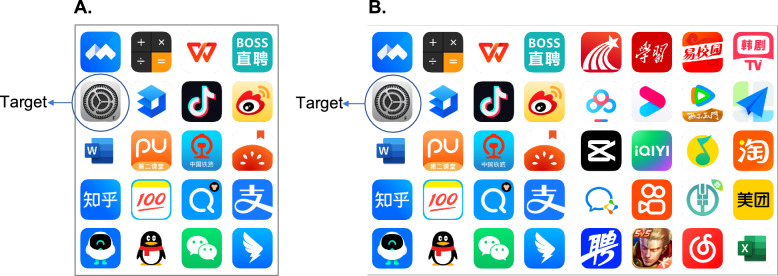
Stimuli in Experiment 2. *Note:* **A** Icons displayed on the small screen. **B** Icons displayed on the large screen, using position-invariant remapping as an example. The circled icon is the search target. Participants were instructed to press the corresponding keys (“F” or “J”, i.e., response targets) shown on the search target

Another group of icons (shown in Fig. 5) used in actual mobile phones (some of them were also used in Experiment 1) was employed in Experiment 2. The steps for making small and large screen images in Experiment 2 were the same as those in Experiment 1.

#### Procedure

The procedure of Experiment 2 is the same as that of Experiment 1, except for the following aspects. (1) Since the greater differences in search performance between the icon remapping were observed in the small-to-large condition in Experiment 1 (i.e., the interaction between Remapping and Transition was significant), we focused on this condition only in Experiment 2. Specifically, we only included small-to-large screen transition trials in this experiment. (2) Experiment 2 additionally included a learning phase before the testing phase. During the learning phase, only the small-screen display was presented, and participants were instructed to search for the target icons, which were randomly shown on 10 out of 20 positions (i.e., positions 1–20 in Fig. 1A). Each target position was repeated twice, resulting in a total of 20 learning trials. In the testing phase, there were 40 trials where participants were instructed to search for the target icon. In 20 of these trials, the target icon was displayed on the learned 10 positions, i.e., the old context, while in the other 20 trials, the target icon was displayed on the unlearned 10 positions, i.e., the new context.

#### Statistical analyses

This experiment employed a 2 (Remapping: order-invariant vs. position-invariant) × 2 (Context: old vs. new) within-subject design. Jamovi 2.3.28 (The jamovi project, 2024) was used to conduct 2 × 2 repeated measures ANOVAs. Response times of all trials were above 200 ms, and therefore, no trials were excluded from the following analysis.

### Results

Similar to Experiment 1, the average accuracies for each condition were all above 96.7%, suggesting that participants completed the task by following instructions. Therefore, the following analysis only focused on RT.

**Fig. 6:**
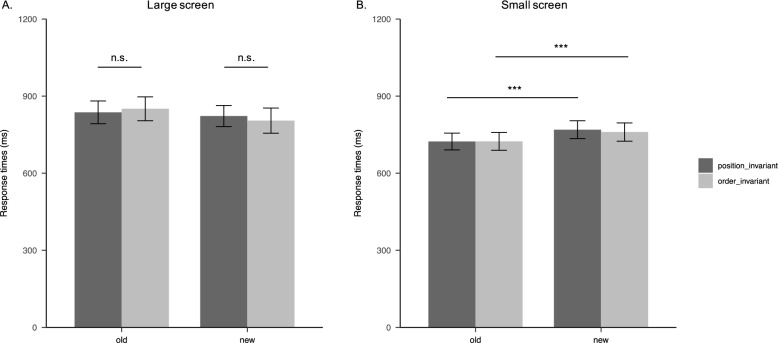
Results in Experiment 2. *Note:* Correct response times for searching target icons on large screens and small screens as a function of Remapping (order-invariant vs. position-invariant) and Context (old vs. new). Error bars denote the 95% CIs, n.s. = non-significant, ***p < 0.001

To inspect whether the contextual cueing effect was observed on large screens, a 2 (Remapping: order-invariant vs. position-invariant) × 2 (Context: old vs. new) repeated-measures ANOVA was performed on RTs of large screens. Results revealed (Table 2; Fig. 6) that the main effect of Remapping was not significant (*F*_1,28_ = 0.012, *p* = 0.912, η_p_^2^ < 0.001). The main effect of Context was significant (*F*_1,28_ = 7.919, *p* = 0.009, η_p_^2^ = 0.220), with shorter RT in the new relative to the old context. The interaction between Remapping and Context was not significant (*F*_1,28_ = 1.699, *p* = 0.203, η_p_^2^ = 0.057). Simple effects analysis further revealed that no significant differences in response times were observed between the two remapping designs in the old context (*t*_28_ = − 0.638, *p*_*b*onferroni corrected_ = 1) and in the new context (*t*_28_ = 0.749, *p*_*b*onferroni corrected_ = 1). Moreover, in the position-invariant condition no significant differences were found between the old and new contexts (*t*_28_ = − 0.922, *p*_*b*onferroni corrected_ = 1), whereas in the order-invariant condition, RT in the old context was marginal-significantly longer than in the new context (*t*_28_ = − 2.768, *p*_bonferroni corrected_ = 0.059). These results indicated that getting familiar with the spatial arrangement of icons in small-screens decreased the differences between the two remapping designs.

**Table 2:** Descriptive statistics of correct RTs in experiment 2 (M ± SD)

Screen size	Remapping method	Old (ms)	New (ms)
Large screen	Position-invariant	837 ± 117	822 ± 109
	Order-invariant	851 ± 122	805 ± 128
Small screen	Position-invariant	723 ± 86	769 ± 91
	Order-invariant	724 ± 91	760 ± 93

Notably, we didn’t found contextual cueing effect on the large screens as expected. To additionally examine whether the contextual cueing effect was observed on small screens, a 2 (Remapping: order-invariant vs. position-invariant) × 2 (Context: old vs. new) repeated-measures ANOVA was performed on RTs of small screens. The results revealed that the main effect of the Remapping was not significant (*F*_1,28_ = 0.134, *p* = 0.717, η_p_^2^ = 0.005); the main effect of Context was significant (*F*_1,28_ = 38.455, *p* < 0.001, η_p_^2^ = 0.579), with response times in the old context being shorter than those in the new context; the interaction between the Remapping and Context was not significant (*F*_1,28_ = 0.786, *p* = 0.383, η_p_^2^ = 0.027). Critically, Simple effects analysis showed that RTs of the old context were shorter than the new context in both the position-invariant condition (*t*_28_ = 4.840, *p*_bonferroni corrected_ < 0.001) and the order-invariant condition (*t*_28_ = 4.769, *p*_bonferroni corrected_ < 0.001). These results suggest that getting familiar with the small screen icon layout can improve search performance for the small screen icons, demonstrating a contextual cueing effect.

### Discussion

Experiment 2 compared search performance between different icon remapping methods in learned (old) and unlearned (new) contexts separately. No significant differences in RTs were found between the remapping methods. These results suggested that learning the spatial arrangement of icons in advance could eliminate the performance differences between the remapping methods.

Moreover, RTs in the old context were longer than in the new context for large screens. Surprisingly, the contextual cueing effect was not observed; instead, an interference effect was found. By contrast, RTs in the old context for small screens were significantly shorter than in the new context, showing a contextual cueing effect. It suggests that learning icon arrangements in small screens can facilitate search speed for small-screen displays. These results together indicate that the contextual cueing effect is more stable only when both the numbers and spatial distribution of target and distractor icons remain constant between the learning and testing phases.

## General discussion

This study investigated the utility of two remapping methods, i.e., position-invariant and order-invariant remapping, in foldable smartphones, as well as the potential roles of contextual cueing in both methods. Experiment 1 revealed that, compared to the order-invariant remapping, participants were faster to locate target icons after the position-invariant remapping, indicating that position-invariant remapping is a more effective design. However, this advantage of the position-invariant remapping was eliminated after participants learned the icon arrangement in the closed state (small screen) in advance, as shown in Experiment 2. These results together suggest that it is likely the contextual effect, i.e., the results of learning icon arrangements, that contributed to better performance for position-invariant remapping in Experiment 1. Moreover, Experiment 2 showed that learning the icon spatial arrangement in the small screen improved search performance for small- but not large-screen icons, suggesting that the contextual cueing effect could be affected by the consistency of numbers and spatial distributions of target and distractor icons between the learning and testing phases.

As expected, we found that position-invariant remapping was a better design for foldable smartphones compared to order-invariant remapping. The key reason for this advantage is that position-invariant remapping preserves most of the spatial configuration between distractors and targets from small screens, allowing the visual system to learn these spatial relationships implicitly (Goujon et al., 2015; Kroell et al., 2019; Sisk et al., 2019). As a result, target locations become more predictable, leading to faster search performance on large screens. In contrast, while order-invariant remapping maintains the horizontal spatial arrangement, it disrupts almost all vertical relationships between icons. This reduces target predictability, weakens spatial contextual cueing effects (Conci et al., 2011; Geyer et al., 2021; Shi et al., 2013), and ultimately results in longer search RTs.

However, the contextual cues learned on the small screen did not facilitate search performance on the large screen after icon remapping. This result was inconsistent with previous studies on tablets (Shi et al., 2013), which found that once spatial contextual cues were established during learning, they continued to improve search performance in the test phase, even when display orientation changed.

A key difference between tablets and foldable smartphones is that screen switches in foldable smartphones involve changes in both icon number and screen size, which means that the absolute positions of target icons change after remapping. Previous research showed that when target locations were relocated, contextual cueing effects often failed to transfer, resulting in search times similar to those in novel displays (Conci et al., 2011; Manginelli & Pollmann, 2009; Zellin et al., 2013). EEG studies also indicated that once a spatial layout was learned, it triggered attentional-priority signals, which may actively misguide attention when the target was relocated (Zinchenko et al., 2020b). These findings suggest that contextual memory is linked to the initially learned target locations, making adaptation to new target positions more challenging rather than entirely flexible.

Notably, extensive practice can eventually enable individuals to adapt to relocated targets, though this process is slow and effortful (Zellin et al., 2014). Therefore, it is critical for foldable smartphones to incorporate onboarding tutorials and training sessions to help users learn and adapt to icon remapping.

Another factor that may influence the magnitude of contextual cueing effects is search strategies. Lleras and Von Mühlenen (2004) found that the passive search strategies (where users rely on learned spatial patterns) led to stronger contextual cueing effects than active search strategies (where users actively explore the display). Additionally, distributed attention, which involves using peripheral vision rather than serial scanning, was found to facilitate faster recovery of contextual cueing after target relocation (Zinchenko et al., 2020a). Future research should control for participants'search strategies to investigate contextual cueing effects more accurately.

Importantly, the performance differences between the two remapping methods observed on the large screen in Experiment 1 were no longer significant following the learning phase in Experiment 2. This added learning phase likely enabled participants to acquire a global spatial context and to internalize the relative positions among icons on the small screen, thereby establishing a stable spatial framework that could support search performance after remapping. Such familiarity may help mitigate the effects of reduced target predictability caused by remapping-induced disruptions in spatial relationships. Recent evidence suggests that when target-context associations become uncertain—as in the case of foldable smartphone transitions—the visual system may shift from relying primarily on target location predictability to engaging alternative mechanisms, such as enhanced distractor suppression (Chen et al., 2024, 2025a). These findings support the view that contextual cueing reflects a dynamic balance between facilitation and suppression processes, underpinned by distinct neural mechanisms (Chen et al., 2025b). Future research should further investigate these mechanisms in the context of foldable smartphone interfaces.

## Future directions

It should be noted that this study relied on behavioral experiments to assess the impact of icon remapping on search performance. Future research may employ eye-tracking technology to examine users’ areas of interest on the screen and attention shifts during the search process, providing deeper insights into visual attention and cognitive mechanisms across different remapping methods. Moreover, the study utilized a limited set of commonly used smartphone application icons as search targets, which may constrain the generalizability of the findings to popular or familiar icons. Future investigations should consider a broader range of icons with varying visual features to assess how different attributes influence search performance under distinct remapping methods. In addition, tri-fold smartphones have recently begun entering the market, introducing greater variations in icon numbers and screen sizes. As a result, the complexity of icon remapping increases significantly in tri-fold smartphones compared to two-fold models. Future studies are needed to investigate how these changes affect usability and to explore optimal icon remapping strategies for tri-fold smartphones.

## Conclusion

In this study, we found that position-invariant remapping offers higher usability compared to order-invariant remapping. Also, pre-learning the spatial arrangement of icons on the small screens during the learning phase increased the search speed for small-screen icons and reduced the performance differences between the two remapping methods on large screens in the test phase. These findings together suggest that position-invariant remapping is the preferred design, as it preserves more contextual cues. This highlights the importance of prioritizing the spatial integrity of the small-screen icon arrangement when designing icon remapping layouts for the expanded large screen.

## Supplementary Information

Additional file 1.

## Data Availability

The data that support the findings of this study are available from the corresponding author upon reasonable request.
