# Supplementary material for: Position-invariant icon remapping facilities search performance in foldable smartphones through the contribution of contextual cueing
Source: Cogn Res Princ Implic. 2025 Sep 1;10:57. doi: 10.1186/s41235-025-00668-9 (PMC12401781; doi:10.1186/s41235-025-00668-9)
Supplement: Supplementary file 1 — Additional file 1. [file 41235_2025_668_MOESM1_ESM.docx]

**Supplementary Materials for:**

**Position-invariant Icon Remapping Facilities Search Performance in Foldable Smartphones through the Contribution of Contextual Cueing**

Supplementary analysis of Experiment 1

To examine whether different remapping methods enhance search performance relative to a non-remapping baseline, we conducted an additional 3 (Remapping: order-invariant vs. position-invariant vs. non-remapping) × 2 (Screen: large vs. small) repeated-measures ANOVA. For the order-invariant and position-invariant remapping conditions, we analyzed search performance after remapping occurred; that is, large-screen performance during small-to-large transitions and small-screen performance during large-to-small transitions, which was the same as the analysis of Experiment 1 in the main text. By contrast, in the non-remapping condition, we analyzed the search performance before any remapping occurred, averaging the pre-remapping performance across all blocks separately for the large and small screens.

Supplementary results of Experiment 1

The 3×2 ANOVA revealed significant main effects of remapping (*F*2,78 = 56.52, *p* < 0.001, η_p_^2^ = 0.592) and screen (*F*1,39 = 251.51, *p* < 0.001, η_p_^2^ = 0.866), but a non-significant interaction between remapping and screen (*F*2,78 = 2.41, *p* = 0.097, η_p_^2^ = 0.058). Post-hoc analysis of the remapping main effect indicated that search times in the position-invariant remapping condition were significantly shorter than both the non-remapping condition (*t*_39_ = -10.12, *p*_Bonferroni corrected_ < 0.001) and the order-invariant remapping condition (*t*_39_ = -7.11, *p*_Bonferroni corrected_ < 0.001). Search times in the order-invariant condition were marginally faster than those in the non-remapping condition (*t*_39_ = -2.32, *p*_Bonferroni corrected_ = 0.076). Thus, these results showed that position-invariant remapping was the optimal approach, outperforming both order-invariant remapping and non-remapped baseline.
